# The impact of research on health education/health literacy on policymaking in Latin America and the Caribbean Region

**DOI:** 10.3934/publichealth.2024017

**Published:** 2024-03-18

**Authors:** Carlos Vílchez-Román, Alberto Paucar-Caceres, Silvia Quispe-Prieto

**Affiliations:** 1 Research Department, CENTRUM Católica Graduate Business School (CCGBS), Pontificia Universidad Católica del Perú (PUCP), Lima 15023, Perú; 2 Department for Operations, Technology, Events and Hospitality Management, Manchester Metropolitan University, Manchester, M15 6BH, United Kingdom; 3 School of Nursing, Faculty of Health Sciences, Universidad Nacional Jorge Basadre Grohmann, Grohmann, Tacna 23000, Perú

**Keywords:** health literacy, education literacy, policymaking, strength of evidence, Latin American countries, mixed-methods research

## Abstract

**Background:**

In this study, we addressed the gap between health research and policymaking in Latin America and the Caribbean (LAC), focusing on health education/health literacy. Despite growing research, translating findings into effective policies needs to be improved. We explored the factors that make research on health education and health literacy to be referenced and mentioned in policy documents in LAC (and in Peru). We proposed a model based on the hypothesis that the relationship between research and policymaking depends on the research strength of scientific evidence, timing, and social media activity.

**Methods:**

A mixed-methods approach was employed, combining quantitative and qualitative data analysis. Quantitative data sources included multidisciplinary databases, altmetric data, and citations of policy documents. For data analysis, we obtained descriptive statistics to identify patterns and then verified the association between variables using χ2. The negative binomial regression was used to test the empirical model introduced above. Quantitative analysis was complemented by analysis of responses to a set of open questions from a sample of Peruvian health policymakers.

**Results:**

We found that timing, strength of evidence, and social media activity were significant predictors of research cited in policy documents. Policy documents tended to rely more on qualitative evidence. A positive correlation between timing and cites in policy documents highlighted the importance of timely dissemination, whereas social media activity, while having an impact, had a relatively minor effect. Peruvian policymakers' responses emphasized the role of political context, the relevance of results, and policymakers' commitment to incorporating research into policies.

**Conclusion:**

Strength of evidence, social media engagement, and publication timing are key predictors of citations for health education/literacy research in LAC policy documents. However, qualitative findings highlight challenges, including some distrust in research findings, together with limited access to relevant research. The findings offer opportunities to enhance evidence-informed health education/health literacy policy decisions.

**Implications:**

To increase the influence on health policymakers, researchers should prioritize the timely dissemination of solid evidence, considering both traditional and digital platforms. Policymakers should focus on the quality and relevance of evidence when formulating policies.

## 1. Introduction

Several authors have explored the gap between research results and policymaking. This gap explains the limited adoption of research results and contribution to evidence-informed policymaking [1],[2]. Concerning health research, in recent decades, there has been a significant increase in the number of research papers focusing on education and health literacy. The expectation was that these studies would inform policymakers and develop effective health literacy policies.

However, there are limitations and gaps in these policies, such as the need for funding, clear goals, and evaluation of policy implementation [3]. For example, the education sector plays a crucial role in achieving health literacy goals, as health literacy can be obtained and used across various settings, including schools [4],[5]. Despite recognizing its importance, health literacy must be more included as an agenda item in education policies [6]. Overall, there is a need for comprehensive and specific health literacy policies that prioritize and operationalize health literacy, allocate resources effectively, and monitor progress and accountability [7],[8].

### 1.1. Problem statement

Researchers have emphasized the importance of understanding the needs of policymakers by translating research into usable formats and improving communication and collaboration between researchers and policymakers to overcome the limited usage of research results in policymaking [9]–[11]. For example, in Innvær et al. [9], one reason for the limited use of research results is the lack of understanding and communication between scholars and decision-makers. To face this challenge, they suggest packaging and presenting research results in formats with structured summaries that are easy to read and understand. Oliver et al. [10] conducted a systematic review of 145 studies. They found that access to information, clarity, relevance, and readability of findings are primary factors limiting the use of research results in health policy.

The authors mentioned above suggest that improved communication and collaboration between researchers and policymakers could enhance the use of scientific evidence in policymaking. Those results align with the findings identified by [12] in their study with US Congress members, where complexity, evidence inconclusiveness, accessibility, presentation, and lack of transparency were identified as barriers. Also, [13] found similar obstacles in their study on Latin American diplomats, policymakers, researchers, and science journalists (paid access to scientific journals, too much and irrelevant information, technical language, or lack of relevance of scientific studies). Therefore, in this study, we aim to answer the following research question: Which factors predict policy document citations at the national or regional levels of health education and health literacy research indexed in the multidisciplinary databases?

### 1.2. Factors that predict the use of results by policymakers

We developed a model underpinned by theoretical, empirical, and methodological strategies to answer the research question. The theoretical rationale identifies factors aligned with the research question that have been previously studied in the literature (e.g., strength of evidence or timing). However, we excluded potentially relevant factors from the model, such as understanding of research results, engagement with an evidence-based approach, or conflicting interests, because we could not obtain this kind of information and include them in the dataset used to validate the empirical model. The empirical evidence examines the relationship between the identified factors, as documented in the literature. Given that this is one of the first studies analyzing factors that predict citations in policy documents, we worked with a convenience sample of the policymakers' use of research results as a proxy of citation. Therefore, we provide indirect evidence supporting the relevance of the selected factors. Finally, regarding the methodological strategies used, we worked with an analytical approach recommended for studies analyzing citations: Negative binomial regression. In the following, we review the supporting empirical model.

Policymakers' use of research results is influenced by several factors that explain the gap between research results and health policymaking in developed and developing countries [14]–[17]. In a study of public healthcare networks of Brazil, Chile, Colombia, Mexico, and Uruguay, researchers found that the lack of institutional support and resources, the willingness of professionals and managers to participate in the policymaking process, and the inadequate working conditions explain the limited inclusion of research results into policy [15]. In China, researchers identified the need to align research with policy and political cycle and the complexity of the policymaking process as primary barriers to translating research into policy [16]. These factors determine whether research findings are considered and incorporated into policy decisions. This study explores the relationship between research citations in policy documents and timing, the strength of scientific evidence, social media activity, and open access status.

Timing is essential for policymakers using research results, who often operate within specific timeframes and deadlines and require timely and relevant information to inform their decisions. Research available and accessible at the right time is more likely to be considered and used by policymakers. However, a significant proportion of policymakers claim that research evidence needs to be delivered at the right time, which can limit its use [18]. The strength of scientific evidence is another important factor. Policymakers value research that is rigorous, reliable, and based on sound methodology. Substantial evidence, supported by robust research methods and findings, is more likely to be trusted and considered in policy development. Policymakers can use research that provides clear and compelling evidence to support specific policy options or interventions [19]–[21]. Social media activity also influences policymakers' use of research results, who increasingly use social media platforms to access and share information. Research widely discussed and shared on social media platforms can gain visibility and reach policymakers who may not have direct access to academic journals or research databases. Social media activity can increase the likelihood of policymakers noticing and considering research [22]. The open-access status of research findings is another significant predictor of their use by policymakers [23],[24]. Open-access research is accessible to anyone, including policymakers, without paywalls or fees. Policymakers often face barriers in accessing research due to limited resources or institutional access and use research findings, increasing the likelihood of incorporating them into policy decisions.

### 1.3. Hypothesis

The strength of scientific evidence, the timing of research results, and the social media activity predict the citation of studies on health education and health literacy in policy documents.

The hypothesis is logically consistent and testable. The logical consistency of the hypothesis lies in the fact that policymakers often rely on scientific evidence to inform their decision-making process [25]. The strength of scientific evidence refers to the quality and reliability of the research findings, which can influence policymakers' trust and use of the evidence [26]–[28]. The timing of research results is essential because policymakers often require timely information to address pressing health issues [18]. Social media activity is relevant because it has become a critical tool for advocating health policy and disseminating research findings [29].

The hypothesis is testable because it can be empirically examined using quantitative and qualitative research methods. Researchers can collect data on the strength of scientific evidence by assessing the methodological rigor, sample size, and statistical significance of studies mentioned in policy documents. The timing of research results can be measured by analyzing the publication dates of studies and comparing them to the dates of policy documents. Social media activity can be quantified by examining the number of mentions, shares, and engagement with research findings on various social media platforms.

In summary, the hypothesis is logically consistent and testable, and it aligns with existing data and facts regarding the use of research evidence by policymakers and the role of social media in health policy advocacy. By examining the strength of scientific evidence, timing of research results, and social media activity, researchers can gain insights into the factors that predict the citations of studies on health education and health literacy in policy documents.

### 1.4. Significance of the study

Originality: This is one of the first models to identify publication-based factors that predict the citation of health education and literacy research results in policy documents. The publication-based factors include timing, strength of evidence, publication status, and social media activity.

Utility: It is a valuable model because policymakers working on public health will know the more robust predictors of citing in policy documents. Therefore, they will be able to understand the dynamics of the stronger predictors. For example, if the strongest one is timing, they can design or implement strategies to be informed about research results applicable to public health.

Scope: This model works mainly for health education and literacy results published in the science mainstream (e.g., journals indexed in the multidisciplinary databases Scopus and Web of Science [WoS]) because most studies analyzed in this work came from both data sources.

## 2. Materials and methods

### 2.1. Study design and approach

We employed a mixed-methods approach with an explanatory design [30]. A mixed-methods approach allows for a comprehensive understanding of the research topic by combining quantitative and qualitative data. This approach is beneficial when studying complex phenomena or exploring the perspectives and experiences of individuals involved in the field of public policy [31]. In the second place, using an explanatory design with the mixed-methods approach can enhance the validity and reliability of the findings. Explanatory design allows for the incorporation of sociological approaches to scholarly research. The research was carried out in two sequential phases. A quantitative component was conducted to 1) examine the gap between research results and citation of these findings in policy documents and 2) identify the predictors of citations in policy documents. Based on these results, a semi-structured interview with open-ended questions was designed to understand why decision-makers from an Andean country incorporate research results in public health policies.

### 2.2. Variables operationalization

Citation in policy documents was quantified as the frequency of citations of a publication with a DOI within policy documents. The frequency counting of citations to a publication with a DOI within policy documents is supported by research demonstrating the importance of quantifying citations in policy documents to understand the broader impact of research [32],[33].

The strength of the scientific evidence was categorized based on the research design, adapting the “evidence pyramid.” The categories ranged from one (case studies and qualitative studies) to five (systematic reviews with meta-analysis for experimental studies) [34]–[36]. Authors of a previous study used the “evidence pyramid” to categorize the strength of the scientific evidence based on the research design. They proposed a new evidence-based practice model for occupational therapy called the “research pyramid” [37]. The timing was operationalized as the time in months between the publication date of a study and its first mention in a policy document. While there may not be a specific study that directly supports this operationalization, it is a logical approach to understanding the temporal relationship between research publication and its impact on policy. Publication status was represented as one for open-access articles and zero for subscription-based articles. Social media activity was assessed by counting the number of tweets received by each study, with retweets being filtered out, but not posts from the same user account, because this last filter had significantly reduced the tweet counting. The assessment of social media activity by counting the number of tweets received by each study is supported by previous research that highlights the use of social media as a source of altmetrics data to measure the impact of research [38].

For the qualitative side, we explored policymakers' adoption of research results. Therefore, policymakers were questioned about factors influencing the adoption of health research findings in policymaking by answering these questions: 1. Why do health policymakers not incorporate research results into their plans and programs? 2. What does it depend on for health policymakers to incorporate research results into their programs? 3. What should scientists and policymakers do to ensure research results are incorporated into programs? Although the qualitative component targeted health policymakers (questions 1–2), scientific opinions were also explored in the study because, at least in public health, sometimes decision-makers were previously scholars or hired scientists as consultants to integrate teams that formulated public policies (question 3). Usually, those policies get published as official or working papers that cite research results in public health. The open-ended questions included topics broader than just the factors predicting citations in health policy documents because health literacy is a relatively less well-known issue among the sample of Peruvian policymakers. In that sense, those questions were included to have a general overview of how health policymakers use health research results.

### 2.3. Data sources and search strategy

For data collection, five distinct sources were utilized. The multidisciplinary databases Web of Science (WoS), Scopus, and The Lens were explored using specific search filters for countries and regions. Multidisciplinary databases provide a comprehensive overview of research conducted in various countries and regions. Those databases are widely recognized and used in bibliometric studies due to their extensive coverage of scholarly literature across different disciplines [39]–[41]. Additionally, altmetric.com was employed to track social media engagement, while Overton was used to capture citations in policy documents. Altmetric.com is a well-known altmetric data provider that captures online mentions and discussions of research outputs on platforms such as Twitter, Facebook, and blogs [42],[43]. Overton registers academic papers' citations in more than 9 million policy documents, providing insights into the research's practical applications and policy relevance [44],[45]. This inclusion enhances the comprehensiveness of this mixed-methods-based study. The study focused on the four indexes within WoS: Science Citation Index Expanded (SCIE), Social Science Citation Index (SSCI), Arts and Humanities Citation Index (AHCI), and Emerging Sources Citation Index (ESCI), and the full indexes of Scopus and The Lens.

The search terms encompassed relevant keywords in the article's title, abstract, or keywords. These terms included “consumer health information,” “health literacy,” and “patient medication knowledge,” combined with “health education.” The search strategies for WoS, Scopus, and The Lens were designed to retrieve pertinent studies. The inclusion criteria involved studies affiliated with Latin American and the Caribbean (LAC) countries-based institutions, while exclusion criteria were not applied based on language, document type, or publication year. Data was collected in August 2023.

### 2.4. Data processing

The records obtained from each database were collated using digital object identifiers (DOIs), a common practice in studies with a similar approach [46]. Following the initial search, we identified 197 records from WoS, 130 records from Scopus, and 97 from The Lens. Duplicate records were removed, resulting in a total of 214 unique DOIs. Removing duplicate records is a standard procedure to ensure the accuracy of the dataset. Subsequently, these DOIs were cross-referenced with Overton and supplemented with data from altmetric.com for the 29 studies (14%) that received citations in policy documents. The collected data were organized and recorded in an MS Excel spreadsheet, then exported as a comma-separated value (CSV) file. Exportation in a standardized format is a common data management and analysis practice.

The sample size in the quantitative analysis was smaller than expected, but it represents all the available data for our study. Initially, we planned to retrieve more studies from the multidisciplinary databases. However, we found less than 220 scholarly works for our research topic: Health literacy/health education in LAC. Given that we used a comprehensive search strategy, the final dataset represents all mentions in health policy documents for the research topic.

Two key variables were incorporated into the dataset: The institution responsible for publishing the policy document containing a study mention (referred to as “citing institution”) and the geographical location of that institution (referred to as “citing country”). Incorporating both variables is essential for analyzing research citing patterns and geographical distribution. Based on alphabetical ordering, numeric identifiers were assigned to each institution (n = 26) and country (n = 26). For instance, the Government of Cuba was assigned the identifier 11, while the Rand Corporation was assigned 22.

### 2.5. Data analysis

The quantitative analysis encompassed descriptive statistics, frequency counts, and correlation matrices. Additionally, χ^2^ tests and Cramer's *V* [47] were employed to explore the association between citations in policy documents, citing institutions, and citing countries. To test the hypothesized model, a Poisson regression model [48],[49] was utilized to predict citations in policy documents using publication status, evidence strength, social media activity, and timing as predictors. Model fit was assessed using R^2^ and the Akaike information criterion (AIC). For the qualitative component, we initially intended to use non-metric multidimensional scaling [50],[51] and exploratory cluster analysis [52] to analyze 16 Peruvian public health policymakers' responses from an emerging topics perspective. However, we did not conduct both exploratory analyses for textual data because of the low % response rate (13%). We carried out descriptive analysis using word clouds and thematic analysis for extracting relevant categories. Instead of using pre-defined categories, we carefully examined the policymakers' responses, considered the purpose of the questions, and extracted categories from an emerging perspective. Initially, we identified patterns and relationships in the textual data, using open and axial coding and comparing responses with questions. This comparison followed an iterative process, manually grouping and regrouping similar texts until we got theoretical saturation and could organize the emerging categories [53]–[55].

Regarding the language used for the qualitative analysis, we used Spanish texts to obtain word clouds because the software program worked with raw text data. However, we translated the original interviewee's responses into English to facilitate reading to report the emerging categories obtained by grouping and regrouping similar texts.

### 2.6. Ethics approval of research

For the quantitative component, we did not require the approval of an institutional review board because no humans were involved as units of analysis. We obtained data from WoS, Dimensions, The Lens, and Overton to test the empirical model. Regarding the qualitative component, the contacted policymakers answered the questions with audio messages shared via WhatsApp and gave their informed consent orally.

## 3. Results

In this section, we present the findings of the two components of the study: Quantitative and qualitative.

### 3.1. Quantitative results

**Figure 1.**
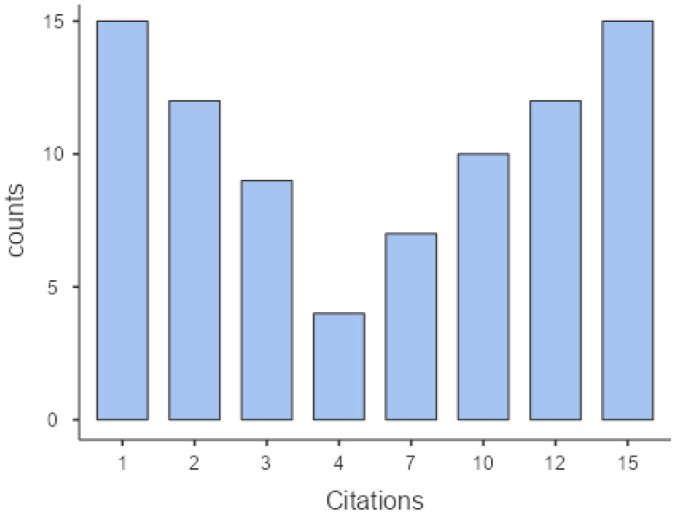
Citations of studies in policy documents.

**Figure 2.**
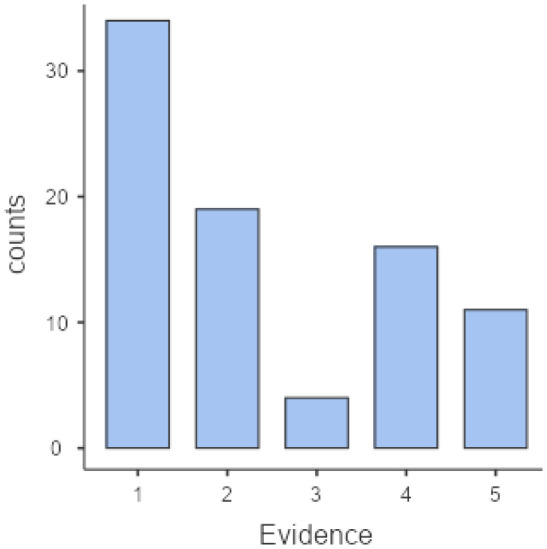
Evidence strength of cited studies.

Citations in policy documents (7.14 ± 5.34 <1–15>) and timing (47.6 ± 33.7 <1–15>) showed a moderate dispersion. Concerning citations, a V-size distribution can be observed for most policy documents, obtaining a significant counting frequency for studies with low <1–2> and high <12–15> numbers of citations. Regarding the strength of evidence, studies with case study and qualitative research design [1], as well as descriptive ones [2] were more frequent within the cited studies than the experimental studies [4] and systematic reviews with meta-analysis [5] (see Figures 1 and 2).

Concerning the quantitative variables, we found a direct statistically significant correlation for timing with citations in policy documents (*r* = 0.531, p < 0.001, IC 95% [0.357–0.669]) and an inverse correlation for timing with activity in social media (*r* = −0.457, p < 0.001, IC 95% [−0.611–−0.269]). Regarding the qualitative variables, we found statistically significant associations between policy document citation and variables included in the model (see Table 1).

**Table 1.** Variables associated with citations in policy documents.

**Variable**	**χ^2^**	**p-value**	**Cramer's *V***
Strength of evidence	202.28	<0.00001	0.776
Publication status	61.70	<0.00001	0.857
Citing institution	239.45	<0.00001	0.638
Citing country	122.94	0.0007	0.457

Concerning the Poisson regression, the strength of evidence, social media activity, and timing appear to be statistically significant predictors of health education and literacy research citations in policy documents. Furthermore, the effect of publication status is not statistically significant (see Table 2). The model explains about 31% of the citation variability estimated by predictors (R^2^ = 0.305). The AIC and deviance values confirm the model's fit: AIC = 552.309 and deviance = 252.335.

Regarding specific effects, holding other predictors constant, a one-unit increase in the strength of evidence (moving from one level on the evidence pyramid to the next higher level) is associated with an approximately 7.02% increase in the expected number of citations in policy documents. The estimate of 0.0135 for timing implies that, on average, for each additional month between the study publication date and the first citation in a policy document, there is a 1.35% increase in the expected number of citations. The highly significant p-value (< 0.001) indicates that this effect is robust and strongly suggests that timing significantly impacts predicting citations. Even though the association with social media activity was statistically significant, its estimate was below 1%.

**Table 2.** Predictors of citations of research on health education and literacy in policy documents.

**Variable**	**Estimate**	**S.E.**	**Confidence interval**	**z-value**	**p-value**
(Intercept)	1.8753	0.0444	95%CI (1.79–1.96)	42.22	<0.001
Strength of evidence	0.0702	0.0276	95%CI (0.02–0.12)	2.54	<0.001
Social media activity	0.0213	0.0065	95%CI (0.01–0.03)	3.30	0.011
Timing	0.0135	0.0014	95%CI (0.01–0.02)	9.98	<0.001

### 3.2. Qualitative results

**Figure 3.**
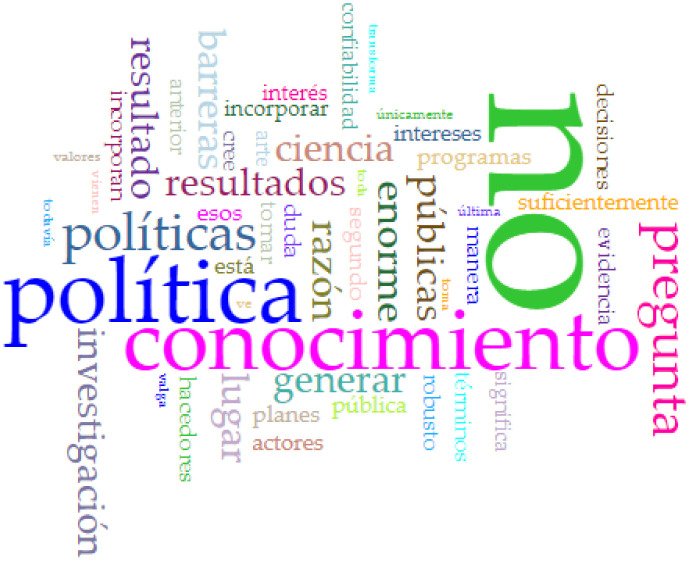
Word cloud of the answer to the question: Why do health policymakers not incorporate research results into their plans and programs?

**Figure 4.**
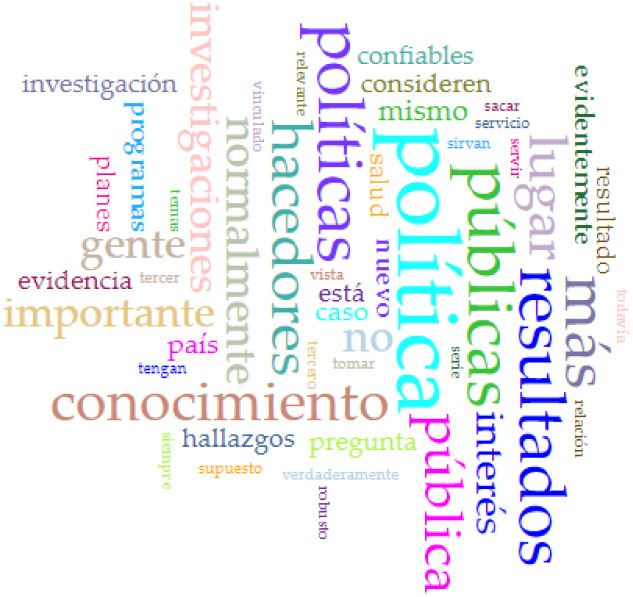
Word cloud of the answer to the question: What does it depend on for health policymakers to incorporate research results into their programs?

**Figure 5.**
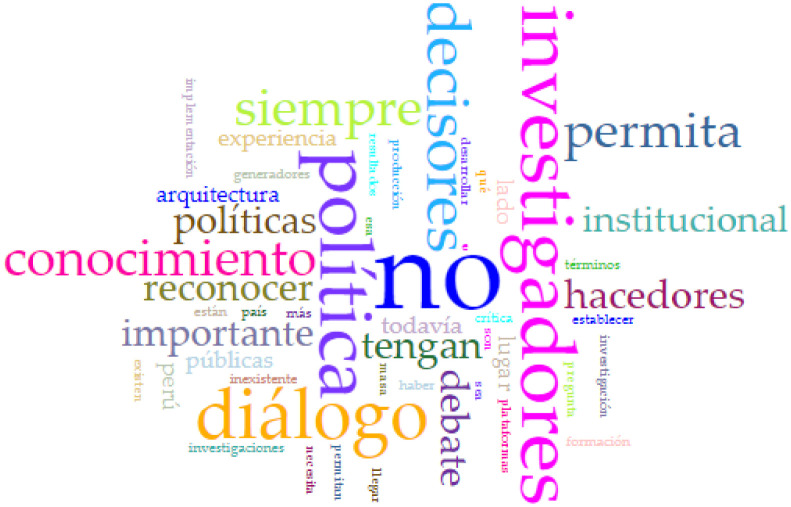
Word cloud of the answer to the question: What should scientists and policymakers do to ensure research results are incorporated into programs?

Given that we received only two responses from the 16 requested, conducting non-metric multidimensional scaling and exploratory cluster analysis was impossible. Therefore, we obtained word cloud plots for the three questions detailed in the previous section. Concerning answers to the first question, Peruvian health policymakers may not incorporate research results into their plans and programs due to barriers related to politics (*política*), a lack of awareness or knowledge (*conocimiento*), and significant policy-related challenges (*políticas*), highlighting the need for addressing these issues to promote evidence-based policy decisions (see Figure 3). According to the Peruvian policymakers' answers to the second question, incorporating research results into health policymaking in Peru depends on factors such as political context (*política*), the relevance of results (*resultados*), and the engagement of policymakers (*hacedores*) in the process (see Figure 4). With relation to the answers to the third question, to ensure research results are integrated into programs, scientists and policymakers should engage in constructive dialogue (*diálogo*) and foster better collaboration, recognizing the importance of research (*investigación*) and the institutional context (*institutional*) in the decision-making process, facilitating the incorporation of evidence into policy (see Figure 5).

We identified seven emerging categories for understanding policymakers' responses in the thematic analysis. For the first question, the categories “untrustworthy results,” “inaccessible results,” and “existing interests” –expressing lack of access and confidence– explained why policymakers do not incorporate research findings in plans and programs. Regarding the second question, the categories “relevance of research findings” and “understanding and engagement” highlighted the primary factors for research utilization in health policymaking. For the third question, the categories “institutionalized dialogue” and "relevant experience” contributed to understanding what decision-makers and scholars need to do to incorporate research findings in health plans and programs (see Table 3).

**Table 3.** Identified categories and quotations for each question.

Question	Category	Interviewee 1 quotation	Interviewee 2 quotation
Why do health policymakers do not incorporate research results into their plans and programs?	untrustworthy results	*“...to be able to implement that knowledge, you have to know it...” “...have an interest in science and believe in science.”*	*“...result.... is not relevant for the moment...” “...doubt about the reliability of the data...”*
	inaccessible results	*“..because they do not have access to, or have no experience in reading or debating, with researchers.”*	*“... the decision maker sees that the road is very paved usually tends to reject the use of this new technology ... because it generates ... additional effort.”*
	existing interests	*“...they have...personal interests, which are not...of the common good...” “...Limitations...on issues of politics, ideology, beliefs, or values.”*	*“...there are powerful economic, social, industrial, corporate interests, that resist incorporating...”*
What does it depend on for health policymakers to incorporate research results into their programs?	relevance of research findings	*“...consider that scientific evidence is fundamental to be able to succeed in public policy...”*	*“...research results are relevant to public policy...reliable” “...make decisions based on meta-analysis...”*
	understanding and engagement	*“...the most important thing from the policymakers' side ... believe in science read, discuss, participate, be linked with those who study these issues.”*	*“...there is an understanding, knowledge, and experience in the country to be able to assimilate the new knowledge...”*
What should scientists and policymakers do to ensure research results are incorporated into programs?	institutionalized dialogue	*“...a fruitful dialogue is needed, a debate with researchers and academics...” “establish platforms that allow this dialogue and this debate.”*	*“...develop an institutional architecture that allows both communities to have a common place where they can dialogue...”*
	relevant experience	*“...researchers themselves have experience in public policy, in implementation...” “...decision-makers have scientific and academic experience ....” “experience from both sides can lead to a useful and fruitful production.”*	*“...a critical mass of researchers and professional policymakers is needed, but large enough to give it a network strength.”*

## 4. Discussion

We aimed to understand the factors influencing the impact of health education and literacy research on policymaking in LAC. Employing a mixed-methods approach combining quantitative and qualitative data, we explored the relationship between research characteristics, social media activity, research timing, and research citations in policy documents. In addition, we sought insights from Peruvian policymakers on research utilization in policymaking. Although it is not the only factor that policymakers would consider, it is recognized that evidence can play a crucial role in political decision-making [56].

Our quantitative analysis reveals that the strength of evidence, social media activity, and timing are significant predictors of citations of research on health education and literacy in policy documents. Specifically, an increase in the strength of evidence is associated with a higher expected number of citations, highlighting the importance of robust evidence in influencing policy decisions [57],[58]. Moreover, social media activity has a positive but relatively small effect on citations, suggesting that while social media can contribute to disseminating research findings, it may not substantially impact policy decisions. Also, timing emerges as a significant predictor, further emphasizing the importance of timely dissemination of research to increase its chances of being included in policy documents.

Qualitative findings, limited by the low response rate, highlight the importance of contextual factors, such as political climate, the relevance of research findings, and policymakers' active engagement in incorporating research into policy. Critical barriers to research utilization include political limitations, insufficient awareness or knowledge, significant policy-related challenges, and existing divergences in public health approaches between researchers and policymakers [59]. Collaborative efforts and constructive dialogue between researchers and policymakers are crucial to facilitating evidence-informed policy decisions [60].

Overall, this study contributes to our understanding of the factors influencing research citations on health education and literacy in policy documents. The findings highlight the need for researchers to prioritize disseminating their findings on time and provide robust evidence to increase the likelihood of their research being incorporated into policy documents.

### 4.1. Contributions to theory and practice

This study contributes to existing scholarship on evidence-informed health policymaking by empirically demonstrating the interplay between research dissemination strategies, strength of evidence, and timing in influencing policy documents. Our findings underscore the significance of disseminating robust evidence through traditional and digital channels to achieve a more significant policy impact. Furthermore, we shed light on LAC policymakers' real-world challenges in translating research into action. The qualitative insights elucidate the need for open dialogue, collaboration between researchers and policymakers, and a nuanced approach to evidence hierarchy in policymaking to bridge knowledge gaps and navigate political complexities.

On a practical level, this research provides actionable insights for both researchers and policymakers. Researchers should prioritize the quality of evidence and timely dissemination, recognizing its substantial impact on policy citations. Policymakers, in turn, should consider the political context, relevance of results, and their active involvement in the process to enhance evidence-informed decision-making.

### 4.2. Contributions to health policymaking

This study holds practical implications for researchers and health policymakers aspiring to create demonstrably impactful policies. It is crucial to consider the incorporation of evidence in this complex decision-making process, which includes logically structured phases, from the problem definition, policy design, implementation, and evaluation of the policy or program impact. Regarding the stakeholders' participation, social actors must reflect on their epistemological and ideological conceptions affecting the interpretation of reality and the problems to be solved [60],[61].

For researchers, it underscores the importance of tailoring communication strategies to reach policymakers and the broader public. Investing in knowledge translation activities and fostering a greater understanding of the local context can help bridge the research-policy gap. For policymakers, focusing on incorporating robust evidence from various research designs and actively engaging with researchers can lead to evidence-informed policies that effectively address health concerns [62]. Investing in knowledge translation resources and establishing clear guidelines for integrating research evidence into policy development are vital to achieving this goal.

## 5. Conclusions

We address the critical gap between health education/literacy research and policymaking in LAC. Our research question sought to identify factors predicting policy citations of health education/literacy research, revealing that evidence strength, social media activity, and timing significantly influence citations in policy documents. Robust evidence emerged as a critical driver, emphasizing the importance of methodological rigor and reliable findings in shaping policy decisions. While positively associated with citations, social media played a relatively modest role, underscoring the need for a multifaceted dissemination approach. Moreover, the qualitative insights from Peruvian policymakers highlighted contextual barriers, including political constraints and challenges, reinforcing the importance of nuanced strategies tailored to local realities. Our mixed methods design empirically validated the hypothesized model and provided rich insights into the complexities of evidence translation into policy. On the quantitative side, the strength of evidence, social media activity, and timing predict citations of LAC research on health education/literacy in policy documents. On the qualitative side, health policymakers do not use research results because of untrustworthy findings and lack of access or relevance.

Researchers are encouraged to prioritize quality and timely dissemination, recognizing the profound impact on policy citations. Policymakers, in turn, should actively engage with researchers, considering the political context and relevance of results. This study contributes to the scholarship on evidence-informed policymaking, providing actionable insights for researchers and policymakers striving for impactful and contextually relevant health policies in LAC.

## 6. Limitations and future research directions

The findings and conclusions reported here must be seen with limitations. The investigation focused on a specific domain (health education and literacy) might not fully capture the dynamics in other health policy areas. Additionally, the limited sample size in the qualitative analysis warrants cautious interpretations of the findings. A broader sample encompassing diverse LAC countries would offer a greater understanding of the regional dynamics surrounding knowledge translation and policymaking.

Further research exploring the effectiveness of specific communication strategies in influencing health policy decisions is crucial [63]. Investigating the role of funding agencies and research institutions supporting evidence-informed policymaking can promote a culture of impactful research translation in LAC.

## Use of AI tools declaration

The authors declare that they have not used Artificial Intelligence (AI) tools in creating this article.
